# Putative Prebiotics Can Disrupt 3D Architecture and Modulate the Microbial Population to Prevent Cariogenic Biofilm Build-Up In Vitro

**DOI:** 10.1021/acsomega.5c11573

**Published:** 2026-05-06

**Authors:** Vinícius Sampaio Alves de Figueiredo, Pedro Canto Bueno, Emilio Alberto Ponce Fuentes, Caroline Dini, Anildo Alves de Brito Júnior, Simone Nataly Busato de Feiria, Renata O. Mattos-Graner, Antônio Pedro Ricomini Filho, Marlise I. Klein

**Affiliations:** † Department of Oral Diagnosis, Piracicaba Dental School, State University of Campinas (UNICAMP), Piracicaba, São Paulo 13414-903, Brazil; ‡ Department of Bioscience, Piracicaba Dental School, State University of Campinas (UNICAMP), Piracicaba, São Paulo 13414-903, Brazil

## Abstract

**Background/Objective(s)/Introduction:** Prebiotics are substances that metabolically favor certain microorganisms of a microbiome, promoting homeostasis. Dental biofilm microorganisms are enmeshed in a matrix of extracellular polymeric substances that they produce. A diet rich in sucrose can lead to a dysbiotic biofilm associated with microbial acid production and a change in the matrix’s composition (mostly water-insoluble glucans), which allows acids to accumulate within biofilms and contribute to teeth demineralization. Thus, the effects of putative prebiotics were evaluated to verify their impact on exopolysaccharides, the microbial population, and biofilm formation. **Materials and methods:** Five potential prebiotics (*N*-acetyl-d-glucosamine, arginine, proline, sodium nitrate, and urea) were evaluated compared with a substance-free control. A *Streptococcus mutans* biofilm model on polystyrene plates was used to determine the concentrations of substances that would inhibit sucrose-derived biofilm formation. Selected concentrations were then used to verify the production of insoluble glucans by glucosyltransferase B. Afterward, *S. mutans* and mixed-species (*S. mutans*, *Actinomyces naeslundii*, and *Streptococcus gordonii*) biofilms were grown on saliva-coated hydroxyapatite discs with sucrose to evaluate the microbial population and 3D biofilm structure (exopolysaccharides and bacterial biovolume). Lastly, a microcosm biofilm formed on polystyrene plates was used to assess the effects of the substances on biomass and the proportion of distinct viable microbial populations. **Results:** Only arginine inhibited insoluble glucan production and *S. mutans* biofilm accretion (≅ 90%). Arginine and proline inhibited a biofilm build-up in mixed-species and microcosm models and modulated microbial counts of species associated with cariogenic biofilms. In the microcosm biofilm, urea hindered biomass accretion in initial biofilms and the counts of aciduric microbiota and fungi, but *N*-acetyl-d-glucosamine stimulated microbial growth. Sodium nitrate affected the size and shape of microcolonies in *S. mutans* and mixed-species biofilms. **Conclusion(s):** Among the substances tested, arginine and proline modulated the microbial population and hindered biofilm accretion, especially arginine, which hampered glucan production. However, urea is the only substance able to impede fungal growth.

## Graphical abstract

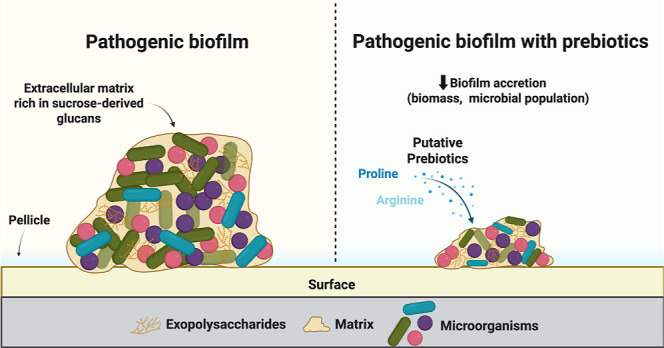

## Introduction

Dental caries remains a significant public health issue worldwide.^1^ This disease results from dysbiosis of the dental biofilm driven by microbial metabolism of dietary carbohydrates. Carbohydrates serve as a substrate for microorganisms to produce extracellular matrix components (chiefly glucans and fructans)^2^ and organic acids. These acids are retained within the biofilm and at the biofilm–tooth interface, and over time, promote tooth demineralization,^3^ leading to the loss of dental structures and harming both oral and systemic health.^4^ This disease has a biopsychosocial origin and results from the frequent consumption of inexpensive dietary sugars and inadequate dental biofilm control, conditions commonly observed in low-income communities.^5,6^ Thus, avoiding sugars would modulate the biofilm microbial community; however, this could be challenging in various social scenarios. Besides, the use of substances that promote the homeostasis of microbial ecology in the oral cavity has been suggested for decades.^7,8^ Hence, the concept of using them as a prebiotic to modulate dental biofilms and prevent dysbiosis is not new; however, incorporating them into oral healthcare products requires more evidence of their effects.

The extracellular matrix can significantly promote dysbiosis of biofilms, where exopolysaccharide producers benefit nonexopolysaccharide producers.^9^ Among the several hundred oral bacterial species, only a few were characterized as exopolysaccharide matrix producers.^10^ Among them, *Streptococcus mutans* is a model organism associated with dental caries^11,12^ because, even at low numbers, it can orchestrate the build-up of cariogenic biofilms when dietary sucrose is available.^13^ This process occurs because the exopolysaccharides produced can encase and protect bacterial cells, allowing them to continue secreting exoenzymes into the milieu including glucosyltransferases (Gtfs) and fructosyltransferase (Ftf). Both Gtfs and Ftf adsorb to dental (in the salivary pellicle) and microbial surfaces. Once surface-adsorbed, these exoenzymes synthesize exopolysaccharides, thereby contributing to biofilm formation.^2^ *S. mutans* Gtfs synthesize water-insoluble glucans rich in α-1,3-linkages (GtfB and GtfC) and water-soluble glucans rich in α-1,6-linkages (GtfC and GtfD), which are critical for assembling a cariogenic matrix.^14^ The insoluble glucans are a virulence determinant because reducing their synthesis affects biofilm cariogenicity, reducing the number and severity of caries lesions.^13^ Moreover, these Gtfs mediate polymicrobial interactions, including those with *Candida* spp., increasing the pathogenic potential of biofilms.^3^

Considering that acids cause tooth demineralization and are retained inside biofilms by exopolysaccharides, substances that could trigger alkali production to counteract acids and/or interfere with exopolysaccharide synthesis and its scaffolding in the matrix, besides selecting microbial species or regulating the load of acidogenic and aciduric ones, would be promising for controlling cariogenic biofilms. The use of prebiotics is one approach being pursued to control cariogenic biofilms.^15^ Arginine has been included as a prebiotic in oral care products because it can be metabolized in alkali that neutralizes acids via the activity of the arginine deaminase system (ADS);^16–18^ however, other substances are being studied.^19,20^ For example, *N*-acetyl- *d* -glucosamine (GlcNAc) and glucosamine (GlcN) amino sugars enhance the antagonistic properties of *Streptococcus gordonii* against *S. mutans* by promoting hydrogen peroxide production and increasing the activity of ADS.^21^ *S. gordonii* more readily metabolizes these substrates as compared to *S. mutans* ^22^ and that alters the proportion of microbial species in the absence of sucrose.^23^ Additional examples shown to modulate oral microbiota and/or oral microbial metabolism are proline,^24^ nitrate,^25,26^ and urea.^26^

There is no information on how these potential prebiotic substances influence the structural traits of cariogenic biofilms, particularly insoluble glucan production and biofilm build-up driven by exopolysaccharide accretion. Therefore, we investigated the effects of substances on the production of insoluble glucan and the structure of cariogenic biofilms using a series of in vitro assays. Our findings showed that each substance affected different properties of biofilms. Arginine was the only substance that markedly impaired glucan synthesis and affected the biofilm 3D architecture and hindered cariogenic microbiota. Proline also affected cariogenic microbiota and biofilm accretion. On the other hand, proline and sodium nitrate interfered with biofilm formation in mixed-species cultures, while urea reduced the proportion of aciduric microbiota (especially fungi) at different phases of the biofilm life cycle.

## Materials and Methods

### Experimental Design

A series of in vitro assays was conducted to investigate the effect of putative prebiotic substances on cariogenic biofilms. First, an *S. mutans* biofilm was formed on 96-well polystyrene plates to determine the concentrations of the substances for downstream assays. Second, the activity of GtfB, the major *S. mutans* exoenzyme synthesizing water-insoluble glucans,^2^ was investigated to verify whether the tested substances affected water-insoluble glucan production, both in solution and when adsorbed. Third, *S. mutans* and mixed-species (*S. mutans*, *Actinomyces naeslundii*, and *S. gordonii*) biofilms were formed on saliva-coated hydroxyapatite discs to determine the influence of the tested substances on biofilm build-up traits (pH of spent medium, bacterial population, biomass, biovolume and architecture). Fourth, salivary microcosm biofilms were formed on 96-well polystyrene plates to determine whether the tested substances altered the proportion of viable aciduric microorganisms, mutans streptococci, and fungi in relation to the total viable microbial populations. The effect of the tested substances on the biofilm biomass was also assessed.

Six groups were assessed, corresponding to different treatments: (1) aminosugar (*N*-acetyl- *d* -glucosamine (GlcNAc)), (2) arginine (considered a prebiotic in oral health care products),^16–18^ (3) proline,^24^ (4) sodium nitrate (NaNO_3_),^25^ (5) urea,^26^ and (6) no prebiotic substance (negative control). The study was approved by the Ethical Committee of the Piracicaba Dental School, State University of Campinas (CAAE: 65533522.4.0000.5418) because human saliva was used to perform in vitro experiments (salivary pellicle and salivary microbiota).

### Putative Prebiotic Substances

The substances tested were purchased from Sigma-Aldrich: (1) GlcNAc (A4106), (2) arginine (A5006), (3) proline (P0380), (4) sodium nitrate (NaNO_3_; S5506), and (5) urea (U5128). All the substances were prepared as stock solutions using Milli-Q water, which were then sterilized (using a 0.22 μm low-protein-binding poly­(ether sulfone) membrane filter).

### Bacterial Strains and Growth Conditions

The strains *S. mutans* UA159 (ATCC 700610), *A. naeslundii* ATCC 12104, and *S. gordonii* DL-1 stocks (at −75 °C in tryptic soy broth plus 20% glycerol) were plated on blood agar plates (37 °C, 10% CO_2_, 48 h). Starter cultures were prepared by inoculating five-ten bacterial colonies into 10 mL of tryptone-yeast extract broth (TY; 2.5% tryptone, 1.5% yeast extract, pH 7.0) supplemented with 1% (w/v) glucose. After incubation (37 °C, 10% CO_2_, 16 h), these cultures were diluted 1:20 in TY + 1% glucose and incubated until the mid-exponential growth phase for the biofilm assays. *Streptococcus milleri* KSB8, carrying a *gtfB* gene from *S. mutans* GS5 and an erythromycin plasmid, was grown as previously described for exoenzyme purification.^28,29^

### Saliva Preparation for Pellicle and Salivary Microbiota

Saliva was donated by nine volunteers (30.9 ± 6.4 years of age) who had not used antimicrobial agents for at least three months. The volunteers rinsed their mouths with 5 mL of Milli-Q water, chewed a parafilm piece, and collected saliva in an ice-chilled Falcon tube. The saliva samples were pooled and diluted 1:1 with adsorption buffer [AB; 50 mM KCl, 1 mM KPO_4_ (0.35 mM K_2_HPO_4_ plus 0.65 mM KH_2_PO_4_), 1 mM CaCl_2_, 1 mM MgCl_2_, 0.1 mM phenylmethylsulfonyl fluoride or PMSF, in dd-H_2_O, pH 6.5].^30^ Saliva was centrifuged (3220*g*, 20 min, 4 °C; Centrifuge 5810R, Eppendorf) and the clarified portion was filter-sterilized (0.22 μm low protein binding poly­(ether sulfone) membrane filter). Saliva was stored at −75 °C until use for pellicle formation. The precipitated portion, which contained the salivary microbiota, was resuspended in 50% glycerol, aliquoted, and stored at −75 °C until use in biofilm assays.^31^

### *S. mutans* Biofilm Formation on Polystyrene Microplates

Biofilms formed by *S. mutans* on polystyrene microplates were used to determine the concentration of the substances for downstream assays. For this purpose, *S. mutans* cultures at the mid-exponential growth phase were diluted in TY + 1% sucrose until they reached a concentration of 2 × 10^6^ CFU/mL. Aliquots of 100 μL of this culture were transferred to 96-well microplates and incubated for adhesion (37 °C, 10% CO_2_, 90 min). The nonadherent cells were removed by aspirating the culture medium and washing the wells with 0.89% NaCl solution once. Next, 200 μL of TY+1% sucrose supplemented with substances (groups 1 to 5) and without substances (group 6) were added to the corresponding wells, followed by incubation (37 °C, 10% CO_2_, 24 h). Uninoculated wells containing a culture medium with and without substances served as controls for each assay. After 24 h of incubation, the microplates were placed on an orbital shaker at 75 rpm for 5 min. Next, the culture medium containing unbound microbial cells was removed, and the remaining biofilms were washed three times with a 0.89% NaCl solution to remove nonadhered cells.

The biomass of the biofilms was assessed using the crystal violet method. Briefly, the washed biofilms were stained with an aqueous solution of 0.1% crystal violet and incubated at 25 °C for 30 min. Next, the wells were washed using Milli-Q water and air-dried (90 min). The crystal violet was eluted with 99% EtOH for 5 min and incubated on an orbital shaker (37 °C, 200 rpm). The eluates were transferred to a new microplate for optical density (OD) readings at 570 nm (plate reader VersaMax 340, Molecular Devices). This assay was performed on three distinct days with four replicates per concentration tested. These concentrations included: 0.4424, 0.2212, 0.1106, and 0.0553% for GlcNAc; 3, 1.5, 0.75, and 0.375% for arginine, proline, and urea; and 0.1105, 0.0553, 0.0276, and 0.0138% for NaNO_3_.

### Analysis of the Effects of Putative Prebiotic Substances on the Activities of GtfB in Solution and Adsorbed to Hydroxyapatite

The enzyme GtfB was purified following previous reports.^28,29^ Briefly, the culture supernatant of strain KSB8 was applied to a chromatography column containing hydroxyapatite beads with buffers containing 0.1 mM PMSF and 0.02% sodium azide (NaN_3_). After purification, the integrity of the enzyme was monitored on an acrylamide gel with silver nitrate staining. Aliquots of GtfB samples were then stored at −75 °C until use. The rationale for testing the enzyme both in solution and adsorbed is that GtfB can be secreted, present in saliva, and adsorbed to teeth and microbial surfaces, as its activity can be distinct when free or adsorbed.^2^

For testing glucan synthesis by fluid-phase GtfB, 50 μg/mL of GtfB in AB buffer with a sucrose substrate (200 mM sucrose, 40 μM 10,200 dextran, 0.04% sodium azide) was incubated with each substance at the tested concentrations. Negative controls included similar samples without GtfB. After incubation (37 °C, mixing, 4 h), 500 μL of 99% EtOH was added, and samples were stored (−20 °C, 18 h) for glucan precipitation. Next, the microtubes were centrifuged (12,000*g*, 30 min), and the pellets were washed three times with 70% EtOH and dried. The glucans produced were solubilized with 200 μL of 1 N NaOH under agitation (37 °C, 2 h). Then, the samples were used for quantifying the glucans.

For testing glucan synthesis by GtfB adsorbed to hydroxyapatite (HA), beads (Macro-Prep Ceramic Hydroxyapatite Type I, 80 μm; Bio-Rad) were coated with saliva for acquired pellicle formation (sHA), as described previously.^30^ Briefly, 2.5 mg of beads per microtube were washed with AB buffer (which contains 0.1 mM PMSF and 0.02% NaN_3_) and then 200 μL of saliva (prepared as described above) was added, followed by incubation (37 °C, mixing, 40 min). Next, the saliva supernatant was removed and the beads were washed three times with AB buffer containing PMSF and NaN_3_. The sHA beads were incubated with a GtfB enzyme (or only AB buffer) by adding 200 μL of GtfB (50 μg/mL) (or AB buffer) to each tube (37 °C, mixing, 40 min). Next, the beads were washed three times with AB buffer (with PMSF and NaN_3_), and 200 μL of substances (at selected concentrations) were added to each tube, followed by incubation (37 °C, mixing, 30 min). Afterward, samples were washed three times with AB buffer (with PMSF and NaN_3_) to remove the substances. Then, 200 μL of sucrose substrate was added to each tube and incubated (37 °C, mixing, 4 h). Next, EtOH was added to stop the reactions, and samples were processed for the quantification of water-insoluble glucans, as described above.

For both fluid-phase and adsorbed GtfB, the quantification of glucans was performed using a phenol-sulfuric acid colorimetric assay with glucose as the standard.^32^ In more detail, the reactions were prepared in clean glass tubes. For the standard curve, aliquots of a 0.01% glucose solution were mixed with 1 N NaOH and Milli-Q water to obtain concentrations of 0.5, 10, 15, 20, and 25 μg, with a final volume of 200 μL. Next, an aliquot of 200 μL of 5% phenol was added to the samples, which were mixed, and then 1000 μL of sulfuric acid (ACS; 95–98%) was added, mixing the samples again. After 20 min at room temperature, the reactions were transferred to cuvettes and read at 490 nm. The standard curve data resulted in a slope of 0.048–0.051 and an *R* ^2^ close to 1. Next, the glucan samples were analyzed.

For the solubilized glucans, an aliquot of 5 μL for samples that were incubated with the enzyme, except for those containing arginine, for which the aliquots were 40 μL, and an aliquot of 40 μL for samples that were not incubated with the enzyme were mixed with Milli-Q water to reach 200 μL. Next, these samples received aliquots of phenol and sulfuric acid, were incubated under the same conditions, and were then read at 490 nm, as for the standard curve points. The values detected for the controls without GtfB were subtracted from the corresponding samples that were incubated with GtfB. The resulting values were converted by using the glucose standard curve used as a reference. Both experiments were performed on three distinct occasions using triplicate samples for each substance tested.

### Single-Species *S. mutans* and Mixed-Species Biofilm Formed on Saliva-Coated Hydroxyapatite Discs

The rationale for using two models was to pinpoint the effects of the putative prebiotics as these effects could be masked in more complex models. *S. mutans* is a model cariogenic bacterium used in the single-species biofilm to pinpoint the contribution of its exopolysaccharide production that shapes the biofilm architecture (especially water-insoluble glucans, but also water-soluble glucans and fructans^3^). As a more complex model, in the mixed-species biofilm, the species can metabolize some of the tested substances, and the metabolites modulate both biofilm build-up and microbial growth, as well as exopolysaccharide accretion. The mixed-species biofilm comprised of *S. mutans*, *S. gordonii* (produces water-soluble glucans, has ADS^33,34^ ), and *A. naeslundii* (has urease activity and reduces nitrate, produces fructans^34–36^ ).

Biofilms were formed on saliva-coated hydroxyapatite (sHA) discs (surface area of 2.93 ± 0.2 cm^2^, Clarkson Chromatography Products Inc., South Williamsport, PA, USA) in batch cultures for 67 h.^30^ Saliva and pellicle preparation were performed as described before.^30^ HA discs were placed vertically in a 24-well microtiter dish using custom-made disc holders.^30^ These discs were incubated with saliva, then dip-washed in AB buffer, and transferred to wells containing either a *S. mutans* culture (for single-species biofilm) or a mixed-species culture for biofilm formation (time 0 h). The bacterial cultures applied were adjusted to 2 × 10^6^ CFU/mL in TY + 0.1% sucrose with and without the tested substances and incubated for 19 h (37 °C, 10% CO_2_), at which point the culture medium was changed. The culture medium was changed twice daily using TY + 0.1% sucrose with and without supplements (at 19 and 43 h) and TY + 1% sucrose with and without supplements (at 27 and 51 h). After each media change, the pH of the spent media was measured. After 67 h of incubation, the biofilms were processed for assessment of microbial counts and biomass (dry weight) or confocal microscopy.

### Biofilm Analysis

#### Determination of Microbial Counts and Biomass (Dry Weight)

At 67 h of development, biofilms were processed for analyses following previously described protocols.^30^ Briefly, each biofilm disc was dip-washed into wells containing a sterile 0.89% NaCl solution (saline solution) and then transferred to a glass tube containing 1 mL of a saline solution. Next, 1 mL of saline solution was used to wash the tube walls, resulting in a total of 2 mL of saline solution for the removal of biofilm from the discs. This process involved placing the glass tubes with biofilms and discs in a beaker and subjecting them to water-bath sonication for 10 min. A sterile metal spatula was used to scrape off any remaining biofilm from the disc surface, and 2 mL of each biofilm suspension was transferred to a new 15 mL tube. Next, each glass tube was washed with 3 mL of saline solution, which was then transferred to the tube containing the initial 2 mL, yielding a total of 5 mL of a biofilm suspension per biofilm/disc. Each biofilm suspension (5 mL) was sonicated using a probe at 7 W for 30 s. Each suspension was diluted (10-fold serial dilution), plated on blood agar plates, and incubated (37 °C, 10% CO_2_, 48 h), to determine the number of CFU per biofilm. For the mixed-species biofilms, the three bacteria present distinct colony morphologies.^37^ The remaining volume was processed to determine the biofilm dry weight, a measure of biofilm biomass.^30^

#### Microscopy Imaging and Computational Analyses of Biofilms

Biofilms were prepared (as described above) with the addition of a 1 μM Alexa Fluor 647-labeled dextran conjugate (absorbance/fluorescence emission maxima of 647/668 nm; Molecular Probes, Carlsbad, CA, USA) to the culture medium throughout the experiment. This was done to incorporate labeled dextran into the exopolysaccharides during synthesis and matrix formation.^14^ When biofilms reached 67 h of development, they were dip-washed in wells containing a 0.89% NaCl solution and then transferred to wells containing saline solution with 2.5 μM SYTO 9 (485/498 nm; Molecular Probes) for visualizing bacteria.^14^ The three-dimensional structure of the biofilms was imaged using a Zeiss LSM 780 confocal laser scanning microscope (Zeiss, Jena, Germany) with a 20× objective lens. Each biofilm was scanned at three randomly selected positions, and a series of images was collected through optical sectioning at each position. The images were analyzed for the 3D reconstruction of exopolysaccharides and bacteria. Additionally, COMSTAT2 was used to quantify the total bacterial and exopolysaccharide content (biovolume), as well as the percentage of coverage per area from the interface disc/biofilm to the top (outer layer) of each biofilm.^14^ ImageJ was used to determine the number of microcolonies and the area occupied by microcolonies in each biofilm evaluated.

### Salivary Microbiota (Microcosms) Biofilm Assay Using Two Polystyrene Microplate Models: Initial Biofilm Formation (24 h) and Preformed Biofilms (48 h)

The inoculum of salivary microbiota was prepared by mixing 1 mL of pre-thawed stock (prepared as described above) with 9 mL of TY + 1% sucrose. A 100 μL volume was transferred per well into two plates: one to determine biomass and the other to count viable microorganisms (using four different agar culture media).

For the analysis of initial biofilm formation, the substances were added immediately after the 90 min adhesion period (time 0 h), and biofilms were evaluated at 24 h of development. For the analysis of preformed biofilms, biofilms were grown for 24 h, then exposed to substances, and their growth was evaluated after 48 h of inoculation. The biomass was assessed by using the crystal violet method. In addition, to determine the viable counts of microorganisms, the biofilms were washed and removed from each well using pipet tips and a 0.89% NaCl solution and then transferred to microtubes. Next, an aliquot of each biofilm suspension was used for a 10-fold serial dilution (10° to 10^–5^) followed by plating on blood agar for total microbiota; acid agar for aciduric microbiota;^38^ Mitis Salivarius Agar with 0.2 U bacitracin and 0.001% potassium tellurite (MSB) for mutans streptococci; and on Sabouraud Dextrose Agar (SDA) with 0.1 mg chloramphenicol for fungi. The plates were incubated for 48 h (37 °C, 10% CO_2_). After incubation, colony counts were performed, and data were analyzed as CFU/biofilm.

### Statistical Analyses

The statistical analyses were performed using Prism 10 (GraphPad Software, Inc. 2024). The Shapiro–Wilk test was used to verify the data distribution (α = 0.05). Data that showed a normal distribution were evaluated with parametric tests, one-way or two-way ANOVA, followed by Tukey’s multiple comparisons test or Dunnett’s multiple comparison test (α = 0.05). Data without normal distribution were evaluated using the nonparametric Kruskal–Wallis test, followed by the Dunn’s multiple comparison test (α = 0.05). The tests used for the data are specified in each corresponding figure caption.

## Results

### Effects of Tested Substances on *S. mutans* Biofilm Formation on Polystyrene Microplates

Figure 1 illustrates the effects of the five tested substances on the biomass of *S. mutans* biofilms formed over 24 h on microplates. Arginine at 3% and 1.5% was the only substance that significantly impaired *S. mutans* biofilm accretion. The 1.5% concentration has been used in previous works^16–18^ and was selected for the subsequent assays. Because the other substances did not significantly affect the biomass, and considering that the third-highest concentration (0.75%) of arginine did not have an effect, and based on data from previous works,^21–26^ 1.5% was chosen for proline and urea, while 0.2212% and 0.0553% were selected for GlcNAc and NaNO_3_, respectively. Of note, the tested substances, at the concentrations selected to verify whether they affected glucan synthesis and/or biofilm growth, do not impair *S. mutans* planktonic growth (data not shown).

**Figure 1:**
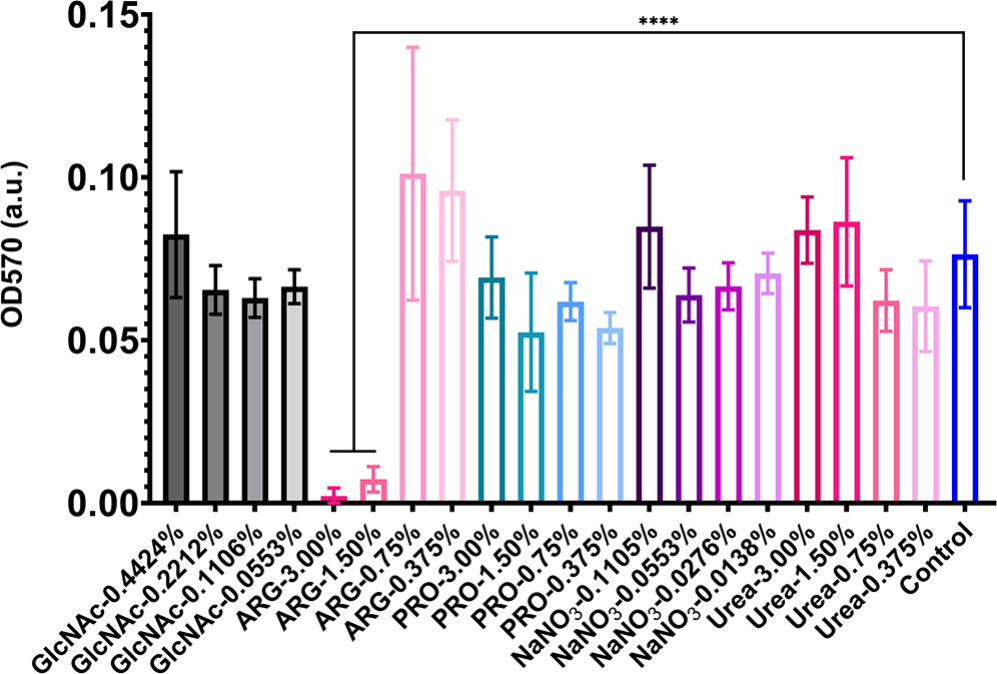
Biomass of *S. mutans* biofilms exposed to distinct concentrations of the tested substances and controls. Biomass was inferred from the crystal violet staining assay by measuring the OD at 570 nm. Data are means, and the error bars correspond to 95% confidence interval (CI) (*n* = 12). The asterisks (****) correspond to *p* ≤ 0.0001 (ANOVA one-way, followed by Dunnett’s multiple comparison test). GlcNAc: *N*-acetyl- *d* -glucosamine; ARG: arginine; PRO: proline; Control: no substance.

### Effect of Tested Substances on Water-Insoluble Glucan Production (GtfB Activity) in Solution and Adsorbed to Hydroxyapatite

As shown in Figure 2, only arginine impaired glucan synthesis in the fluid-phase and adsorbed GtfB, which is consistent with the *S. mutans* biofilm formation profiles (Figure 1). The other substances had no marked effect on the exoenzyme activity.

**Figure 2:**
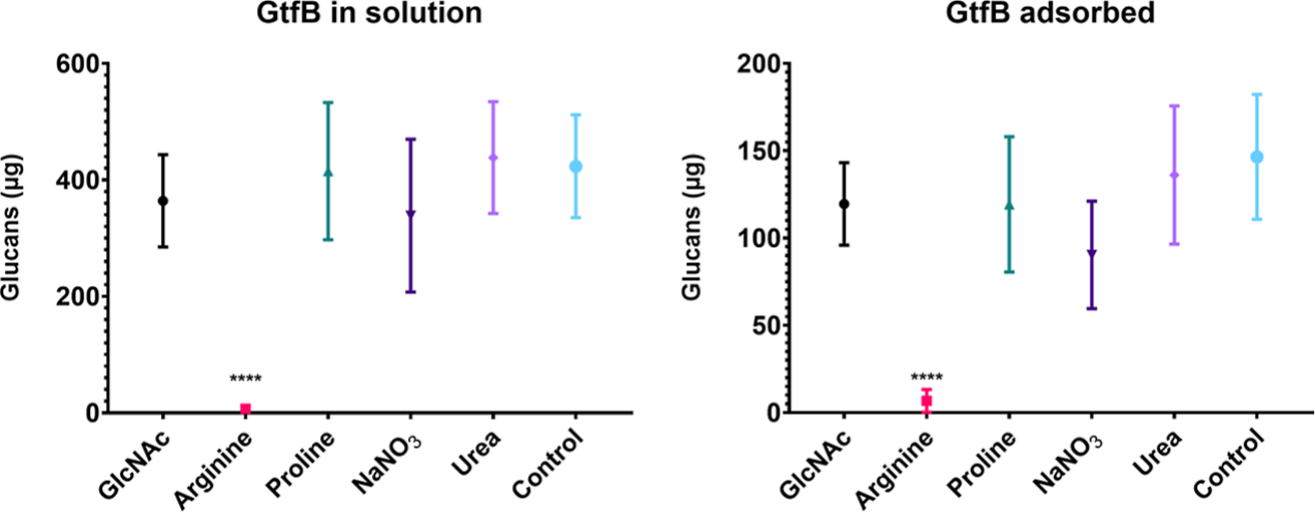
Effect of tested substances on water-insoluble glucans produced by GtfB in solution and adsorbed to saliva-coated hydroxyapatite. Data are means of glucans produced (μg), and the error bars correspond to 95% CI (*n* = 9). The asterisks (****) correspond to *p* ≤ 0.0001 (ANOVA one-way, followed by Tukey’s multiple comparison test). Control: no substance.

### Effects of Tested Substances on Cariogenic Traits of Single-Species *S. mutans* and Mixed-Species Biofilms Formed on Saliva-Coated HA Discs

Figures 3 and 4 show the effects of the putative prebiotics on the profiles of pH (measured on a spent medium), biofilm dry weight, and microbial counts of *S. mutans* and mixed-species biofilms formed on saliva-coated HA discs. Arginine was the only substance that impaired pH drop values in *S. mutans* and mixed-species biofilms compared to the control (Figure 3A,B); while GlcNAc promoted significant pH reduction in mixed-species as compared to the control (Figure 3B), indicating that this aminosugar could have been metabolized in higher amounts of acids by the bacteria in this model or that the acids produced by them did not become trapped inside the biofilms.

**Figure 3:**
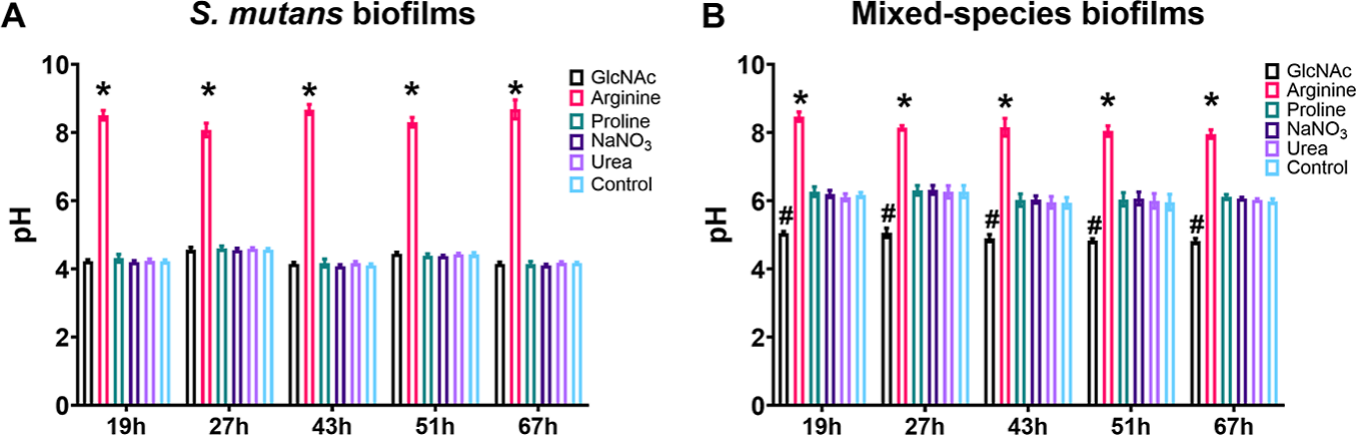
Effect of tested substances on pH of spent media in *S. mutans* (A) and mixed-species (*S. mutans*, *S. gordonii*, and *A. naeslundii*) (B) biofilms. Data are means, and the error bars correspond to 95% CI. The asterisk (*) represents *p* ≤ 0.05 for arginine versus all other groups, while the number sign (^#^) represents *p* ≤ 0.05 for GlcNAc (ANOVA two-way, followed by Tukey’s multiple comparison test). Control: no substance.

**Figure 4:**
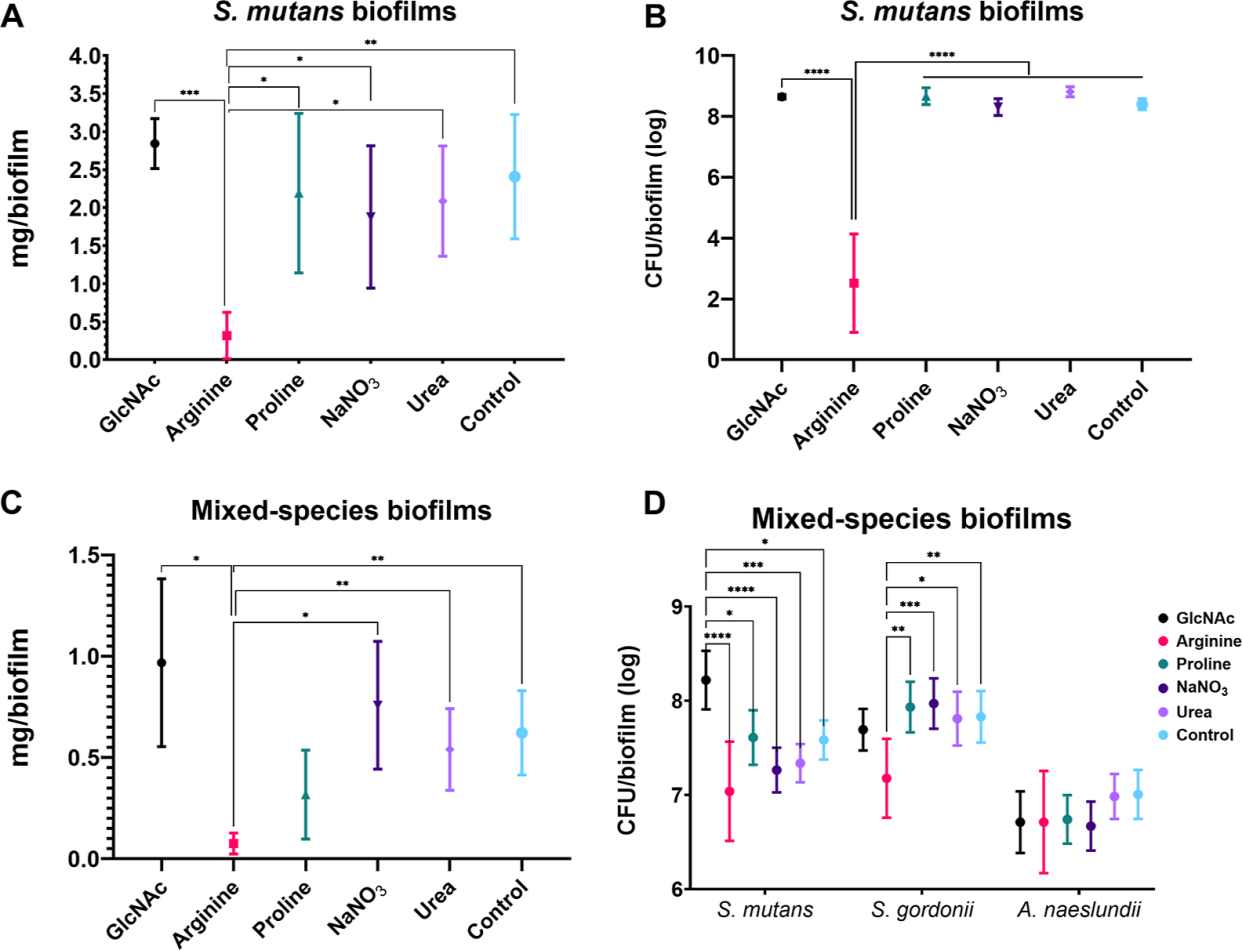
Effect of tested substances on *S. mutans* and mixed-species (*S. mutans*, *S. gordonii*, and *A. naeslundii*) biofilms. The figure depicts the viable bacterial population (CFU/biofilm), and the biomass (dry weight: mg/biofilm). The upper graphs represent the data from *S. mutans* biofilms (A,B), while the bottom graphs represent the data from mixed-species biofilms (C,D). Data are means, and the error bars correspond to 95% CI. The asterisk represents statistical difference for depicted comparisons (****p ≤ 0.0001; ***p ≤ 0.001; **p ≤ 0.01; *p ≤ 0.05; ANOVA one-way, followed by Tukey’s multiple comparison test for (A–C); ANOVA two-way, followed by Tukey’s multiple comparison test for (D)). Control: no substance.

In the single-species biofilms, arginine was also the only substance that clearly hindered biofilm biomass, as measured by its dry weight and *S. mutans* counts (Figure 4A,B). Moreover, arginine strongly inhibited the biomass accretion of mixed-species biofilms and the counts of *S. mutans* and *S. gordonii* (Figure 4C,D). Proline also hindered biomass of mixed-species biofilms (Figure 4C), whereas it did not significantly affect the counts of *S. mutans* or other species in these biofilms (Figure 4D). Of note, GlcNAc promoted lower pH values and higher *S. mutans* counts in mixed-species biofilms (*p* ≤ 0.05; two-way ANOVA, followed by Tukey’s multiple comparison test).

### Effects of Tested Substances on 3D Architecture and Quantity of Bacterial and Exopolysaccharide Biovolumes in the Single- and Mixed-Species Biofilms

The 3D structure of single-species *S. mutans* biofilms formed during 67 h (Figure 5) was markedly different from that of the mixed-species biofilms (Figure 6) and was differently affected by each of the tested substances (e.g., the size and morphology of microcolonies). These differences were further reflected in the measures of bacterial and exopolysaccharide biovolumes (Figure 7), in the proportions of exopolysaccharides and bacteria as a percentage of the area covered by bacteria and exopolysaccharides (Figures 8 and 9), and in the counting of microcolonies and the area occupied by them in the two biofilm models (Figure 10).

**Figure 5:**
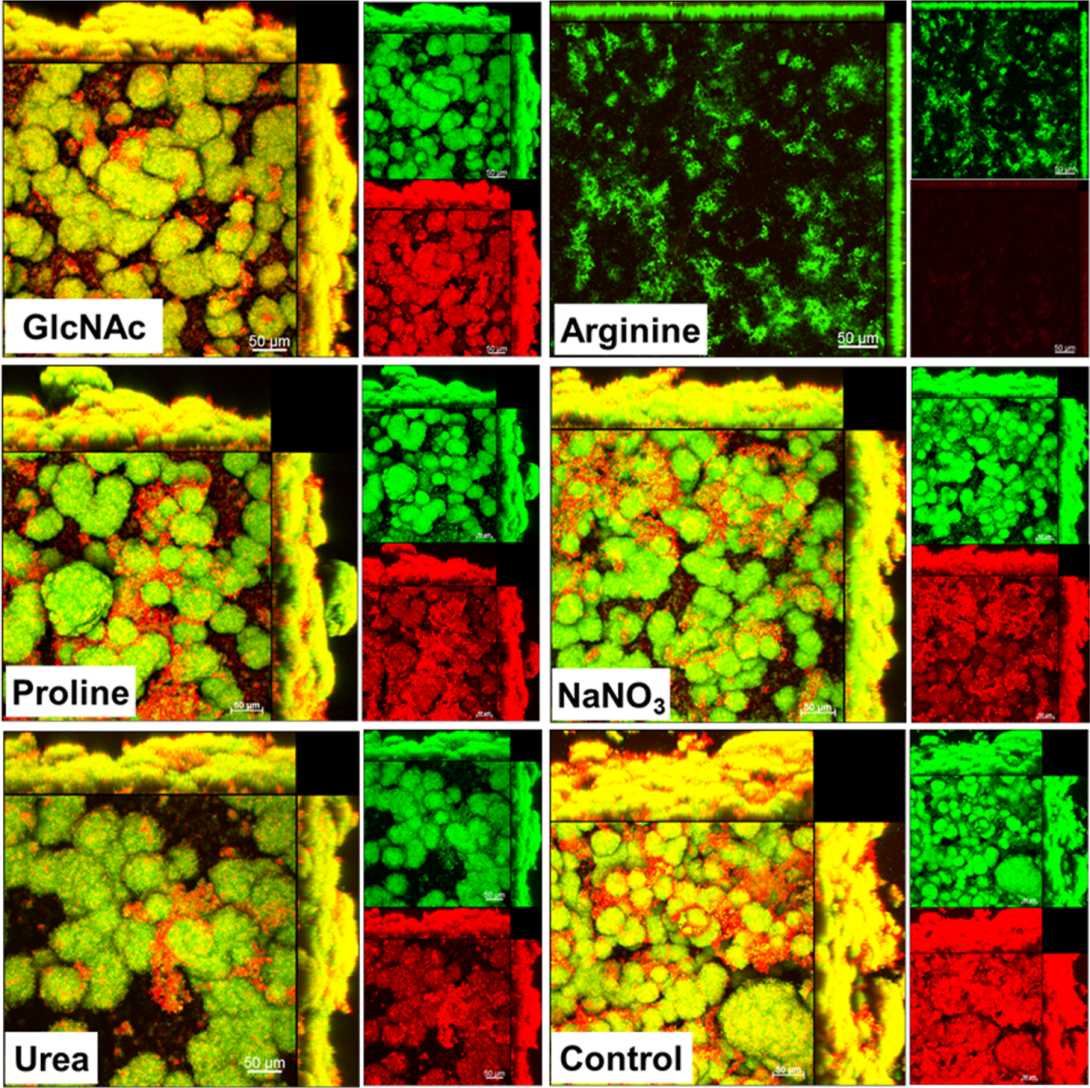
Architecture of *S. mutans* biofilms exposed to tested substances. Representative 3-D renderings of biofilms at 67 h of development. Green represents bacterial cells labeled with SYTO 9, and red represents the exopolysaccharides stained with Alexa Fluor 647-labeled dextran conjugate. The imaging was performed using a Zeiss LSM 780 AxioObserver microscope equipped with a 20×/0.5 objective lens. The larger image in each set represents the overlaid red and green channels. The scale bar corresponds to 50 μm. Control: no substance.

**Figure 6:**
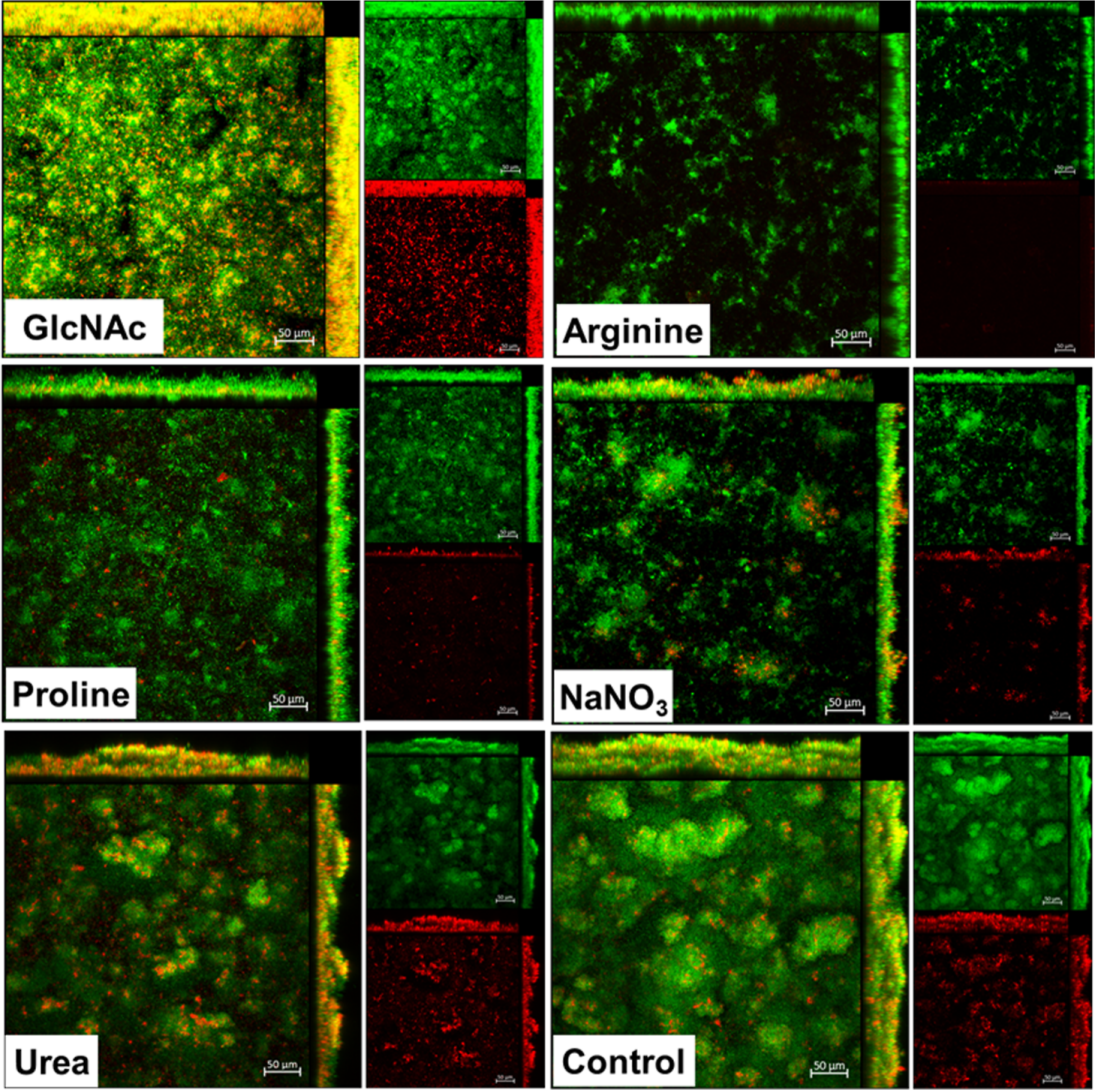
Architecture of mixed-species biofilms exposed to tested substances. Representative 3-D renderings of biofilms at 67 h of development. Green represents bacterial cells labeled with SYTO 9, and red represents the exopolysaccharides stained with Alexa Fluor 647-labeled dextran conjugate. The imaging was performed using a Zeiss LSM 780 AxioObserver microscope equipped with a 20×/0.5 objective lens. The larger image in each set represents the overlaid images of the red and green channels. The scale bar corresponds to 50 μm. Control: no substance.

**Figure 7:**
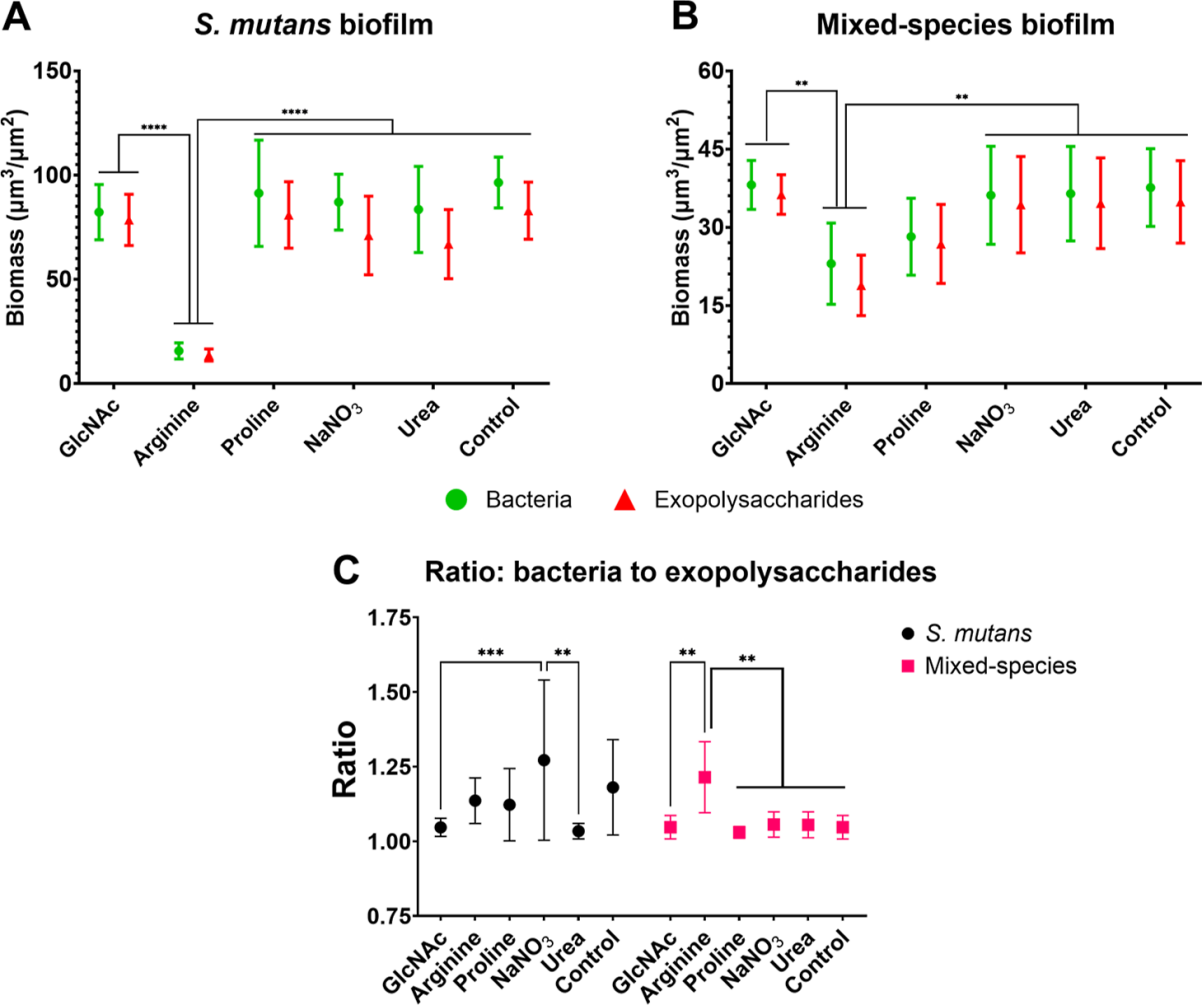
Biovolume of exopolysaccharides and bacteria in *S. mutans* single-species and mixed-species biofilms exposed to the tested substances. Biovolumes are represented as the biomasses (μm^3^/μm^2^) of bacteria and exopolysaccharides for *S. mutans* (A) and mixed-species (B) biofilms at 67 h. These data were used to determine the ratio of bacterial biomass (biovolume) to exopolysaccharides (C). The plotted data represent averages, and error bars indicate 95% CI (*n* = 6 per biofilm). The asterisk represents statistical difference for depicted comparisons (****p ≤ 0.0001; ***p ≤ 0.001; **p ≤ 0.01; ANOVA two-way, followed by Tukey’s multiple comparison test). Control: no substance.

**Figure 8:**
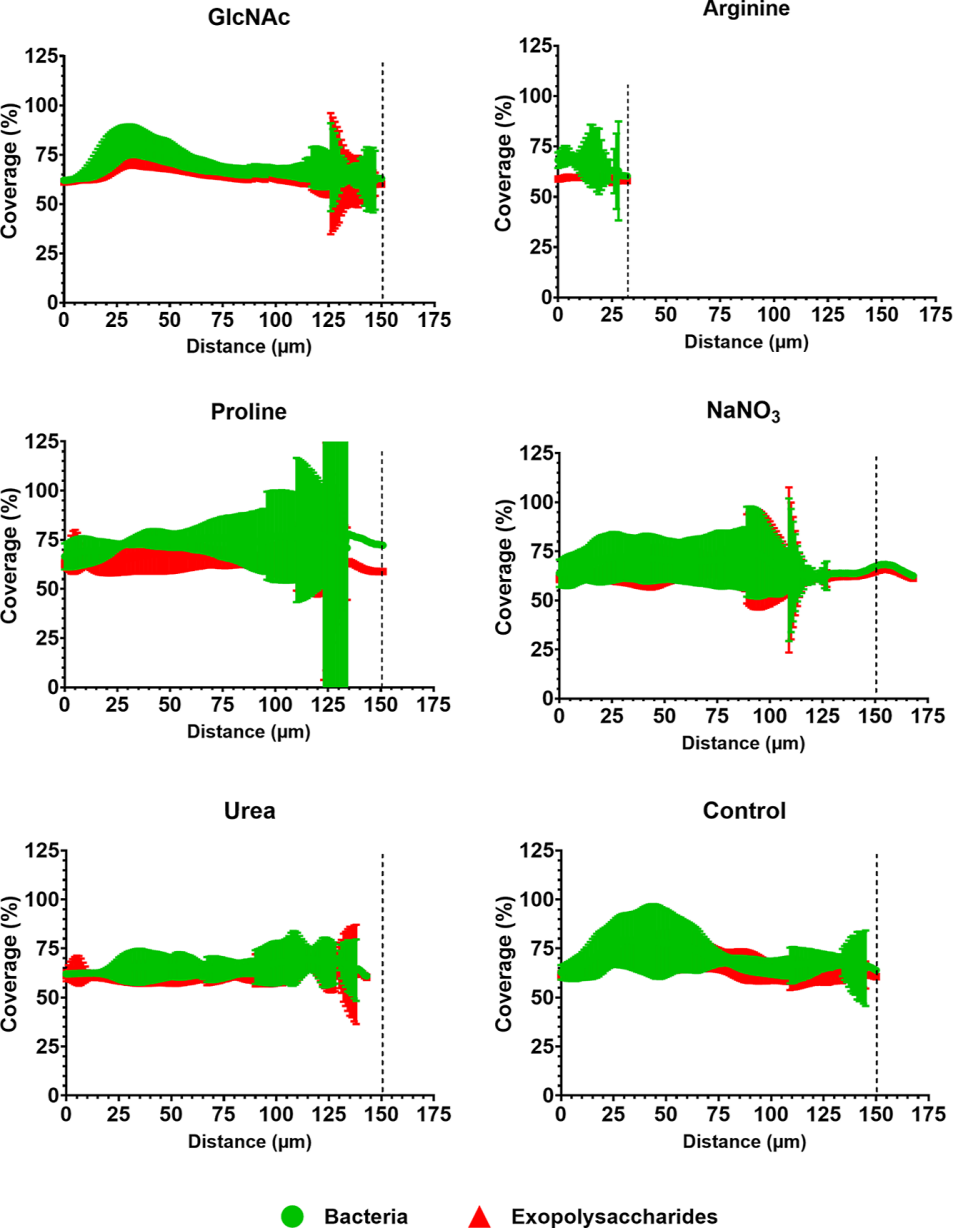
Profiles of the distribution of exopolysaccharides and bacteria in *S. mutans* single-species biofilms exposed to tested substances. The data represent the mean percentage of coverage per area, ranging from the interface of the HA disc-biofilm to the outer layer of each biofilm at 67 h (error bars indicate 95% CI; *n* = 6 images per biofilm). The vertical line serves as a guide to compare the tested substances with the control, which contains no substance. Control: no substance.

**Figure 9:**
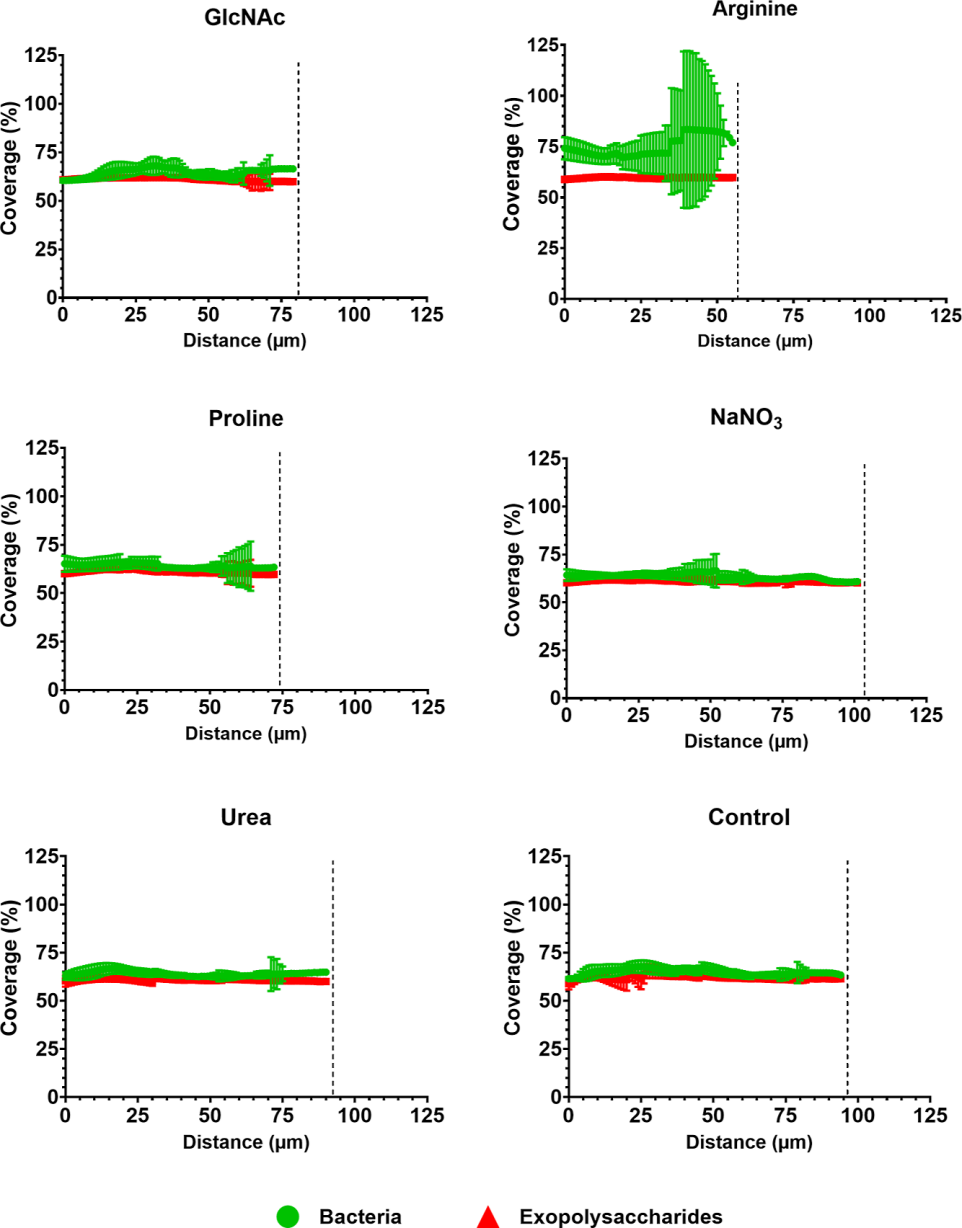
Profile of the distribution of exopolysaccharides and bacteria in mixed-species biofilms exposed to tested substances. The data represent the mean percentage coverage per area, ranging from the interface HA disc-biofilm to the outer layer of each biofilm at 67 h (error bars indicate 95% CI; *n* = 6 images per biofilm). The vertical line serves as a guide to compare the tested substances with the control, which contains no substance. Control: no substance.

**Figure 10:**
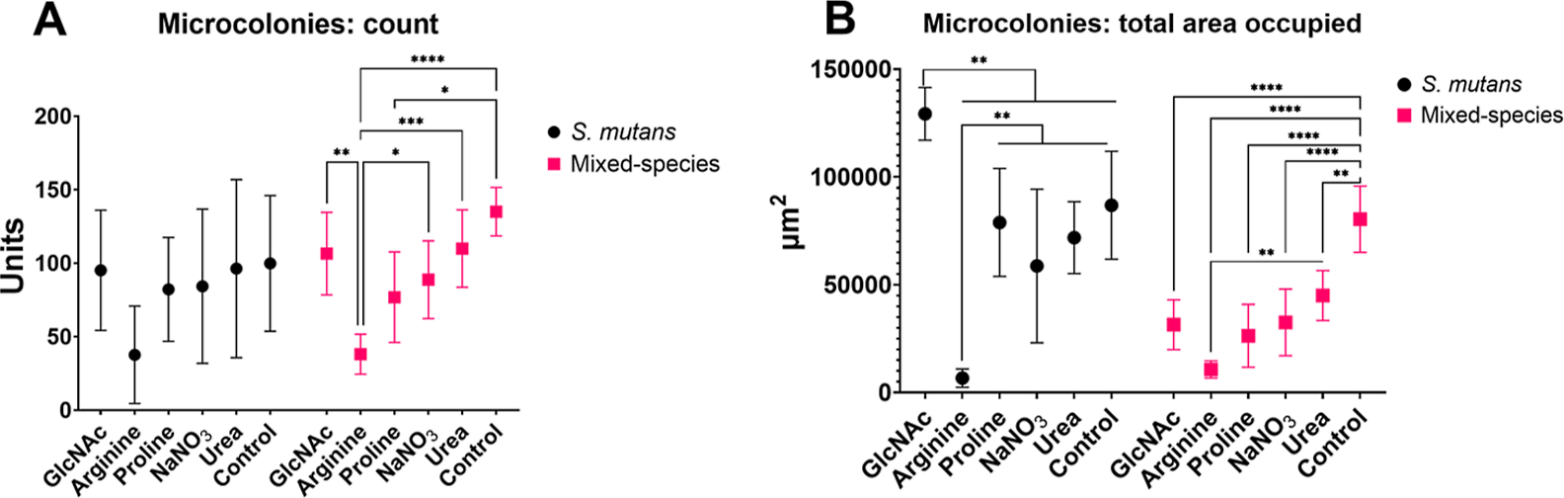
Microcolonies count (A) and total area occupied (B) in *S. mutans* single-species and mixed-species biofilms exposed to tested substances. The plotted data represent averages of counts and occupied area of biofilms at 67 h, and error bars indicate 95% CI (*n* = 6 per biofilm). The asterisk represents statistical difference for depicted comparisons (****p ≤ 0.0001; ***p ≤ 0.001; **p ≤ 0.01; *p ≤ 0.05; ANOVA two-way, followed by Tukey’s multiple comparison test). Control: no substance.

Specifically, *S. mutans* biofilms exposed to all substances, except arginine, and the negative control showed well-defined microcolonies of bacterial cells enmeshed in an exopolysaccharide matrix, although the sizes of microcolonies were variable (Figure 5). In *S. mutans* biofilms exposed to arginine, exopolysaccharides are visually absent, clearly differing from those exposed to other substances. On the other hand, mixed-species biofilms exhibit less-defined microcolonies, although the control group showed larger microcolonies than the other groups. In mixed-species biofilms, the microcolonies mostly resemble spread-out cell clusters, not concise and round-shaped ones seen in *S. mutans* biofilms (especially for the sodium nitrate group). Consistent with the effects of proline on the dry weight (Figure 4C), this substance also clearly hindered the biovolume of exopolysaccharides (Figure 7) and in the cell clusters in the mixed-species biofilms (Figure 6). Additionally, NaNO_3_ affected the overall size of the microcolonies in *S. mutans* biofilms, particularly in the mixed-species biofilms.

The biovolume and coverage data of our microscopy analysis (Figures 7–9) were further consistent with our measures of dry weight of single- and mixed-species biofilms, confirming that arginine and proline reduced biofilm formation in the mixed-species models, whereas only arginine affected biofilm formation by *S. mutans* in the single-species model. In addition, the coverage data for exopolysaccharides and bacteria (Figures 8 and 9) corroborate the quantitative biomass data (Figure 7) and the overall structural organization observed in Figures 5 and 6. Of note, the ratio of bacterial biomass (biovolume) to exopolysaccharides was close to 1 for most biofilms, with slight differences (Figure 7C). *S. mutans* biofilms exposed to nitrate showed a higher ratio of bacteria to exopolysaccharides than those exposed to GlcNAc or urea, indicating that there were more bacteria than exopolysaccharides in the former. In contrast, in mixed-species biofilms, those exposed to arginine showed a higher ratio than those in all other groups.

In addition, Figure 10 shows that the quantity of microcolonies did not differ statistically for *S. mutans* single-species biofilms; however, the area occupied by these microcolonies did differ markedly, as GlcNAc yielded a larger area occupied while arginine yielded a meager area (versus all other groups). In contrast, for mixed-species biofilms, the quantity of microcolonies was lower for the arginine group than for the other groups, except for the proline group. In these mixed-species biofilms, the area occupied by microcolonies was larger for the control group than for all other groups. Thereby, a comprehensive evaluation of the biofilm 3D structure coupled with quantification of its traits demonstrates that the tested substances indeed exert an effect on biofilms, depending on the model used.

### Effects of the Tested Substances after Treatment of Adhesion-Phase and 24 h-Old Microcosm Biofilms

Figure 11 shows the effects of the tested substances, added to surface-adherent microorganisms (Figure 11A) or to preformed 24 h-old biofilms (Figure 11B), after 24 h of incubation for microcosm biofilm development. During initial biofilm formation (24-h-old biofilms exposed from 0 h to the substances), arginine and urea hindered biofilm build-up (Figure 11A); in contrast, when preformed biofilms were exposed 24 h onward to the substances, proline was effective in impeding further biofilm accretion (Figure 11B).

**Figure 11:**
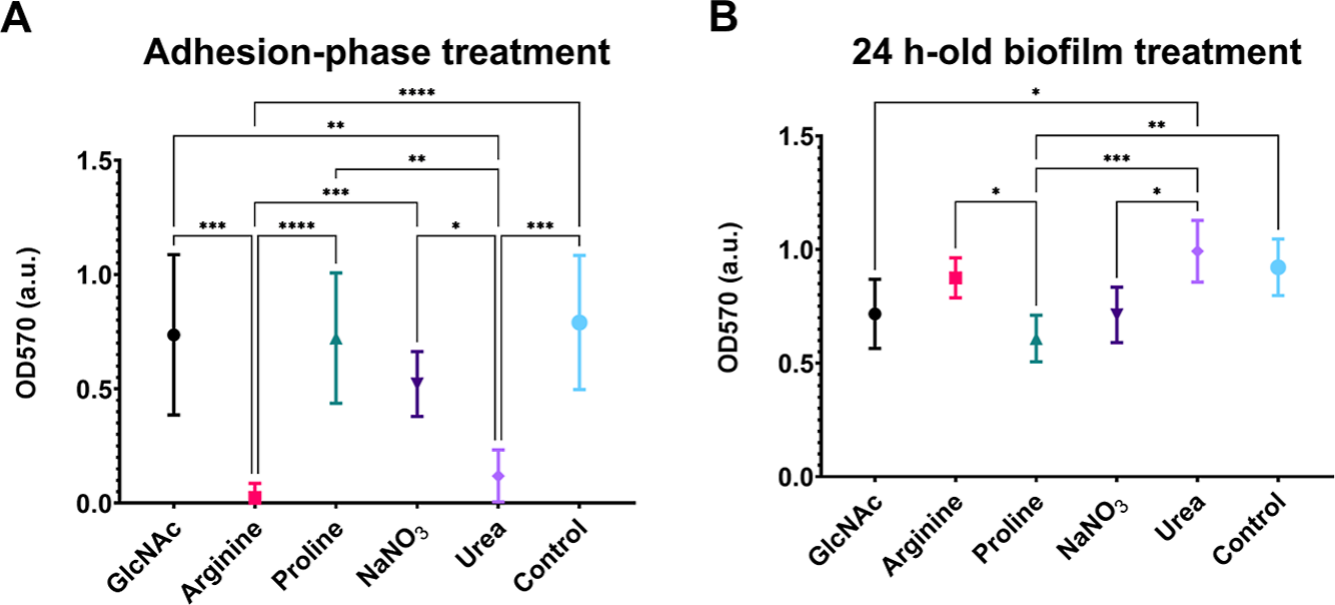
Biomasses of adhesion-phase treatment (A) and 24 h-old biofilm treatment (B) of microcosm biofilms obtained via the crystal violet method. Data are means, and the error bars correspond to 95% CI. The asterisk represents statistical difference for depicted comparisons (****p ≤ 0.0001; ***p ≤ 0.001; **p ≤ 0.01; *p ≤ 0.05; for (A) Kruskal–Wallis test, followed by Dunn's multiple comparison test; for (B) ANOVA one-way; followed by Tukey’s multiple comparison test). Control: no substance.

Besides the biomass, the number of different viable microorganisms of treated biofilms (from initially forming or from preformed biofilms) was also compared (Figures 12 and 13). As shown in Figure 12, urea mainly affected the proportion of aciduric microorganisms and, consistently, of fungi. On the other hand, its effects on the counts of mutans streptococci and total microbiota were limited. These effects were further observed when urea was added to preformed 24 h-old microcosm biofilms (Figure 13). In addition, arginine showed a dominant effect on the viable counts of mutans streptococci, but limited effects on counts of fungi and total aciduric microorganisms when added to forming and preformed biofilms. In contrast, GlcNAc, proline, and nitrate did not markedly affect the counts of the evaluated microbiota, although slight differences were observed (Figures 12 and 13). Thus, in a microcosm biofilm model, the biofilm build-up was impeding biomass and specific microorganisms were observed primarily for arginine, and the magnitude of this effect depends on whether it is introduced during initial or preformed biofilm. Moreover, proline impeded the build-up of biomass when introduced into preformed biofilms but did not markedly affect microbial counts, indicating that its effect may be linked to matrix structuring.

**Figure 12:**
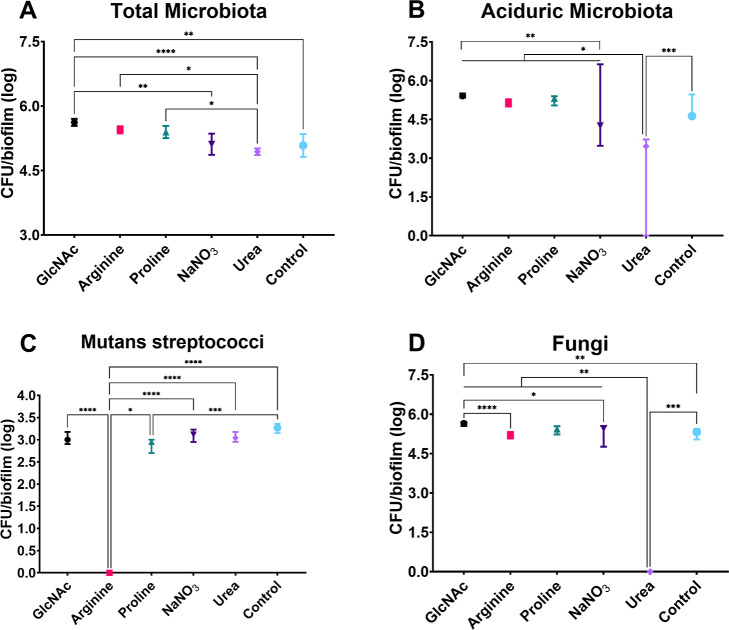
Microbial population of adhesion-phase treatment of microcosm biofilms. Distinct microbial populations were total microbiota (A), aciduric microbiota (B), mutans streptococci (C), and fungi (D). Data are means, and the error bars correspond to 95% CI. The asterisk represents statistical difference for depicted comparisons (****p ≤ 0.0001; ***p ≤ 0.001; **p ≤ 0.01; *p ≤ 0.05; Kruskal–Walliś test, followed by the Dunn’s multiple comparison test). Control: no substance.

**Figure 13:**
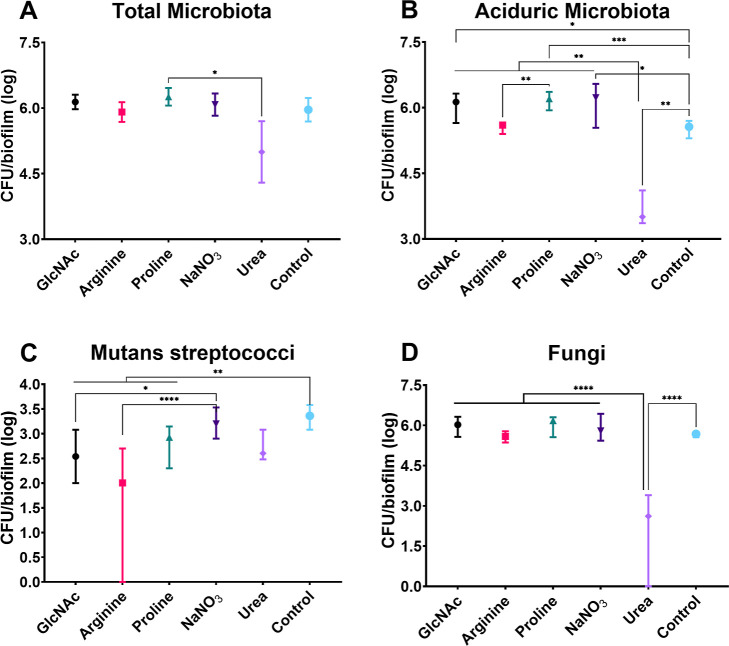
Microbial population of 24 h-old biofilm treatment of microcosm biofilms. Distinct microbial populations were total microbiota (A), aciduric microbiota (B), mutans streptococci (C), and fungi (D). Data are means, and the error bars correspond to 95% CI. The asterisk represents statistical difference for depicted comparisons (****p ≤ 0.0001; ***p ≤ 0.001; **p ≤ 0.01; *p ≤ 0.05; Kruskal–Wallis' test, followed by Dunn's multiple comparison test). Control: no substance.

## Discussion

The use of prebiotic substances that could impede biofilm build-up and promote microbial shifts toward a beneficial, less acidogenic, and aciduric microbiota emerges as a promising approach for controlling biofilm cariogenicity, especially in the presence of dietary sucrose. Here, the data support the putative prebiotic effects of proline for controlling cariogenic communities in mixed-species and microcosm biofilms. Moreover, our data confirm reports on the effect of arginine, a prebiotic substance used to control dental biofilms. Arginine was shown to inhibit the expression of the *S. mutans* *gtfB* gene,^39^ and it was postulated that the effect observed on *S. mutans* biofilms may be due to arginine interfering with Gtfs activity.^40^ This hypothesis was supported here because we demonstrate that arginine impairs GtfB activity both in the fluid-phase and when adsorbed to HA surfaces, which further explains its significant effects on cariogenic biofilms.

Although none of the substances tested had an effect similar to that of arginine in impeding glucan synthesis, all the tested substances affected biofilm communities and/or structure in a specific fashion. For instance, the tested substances could have interfered with glucan binding, a process critical for the 3D architecture of sucrose-derived cariogenic biofilms.^2,41^ This hypothesis is supported by the altered morphology and size of microcolonies, for example, by sodium nitrate in *S. mutans* single-species and mixed-species biofilms, or by urea, which hindered the counts of aciduric microbiota and fungi that contain several microorganisms to which Gtfs adsorb and synthesize glucans;^3^ so if these microbial surfaces are less abundant, the bridging and glucan binding will be altered, possibly affecting biofilm’s cariogenicity. In this context, given that the ratio of bacterial biomass (biovolume) to exopolysaccharides was close to one for most biofilms, with slight differences; with *S. mutans* and mixed-species biofilms exposed to arginine having a higher ratio (versus others) and that previous studies have proven that a lower ratio of bacteria to exopolysaccharides indeed affect diffusion and creates acidic microniches,^14^ changes in this ratio, even, small ones could be meaningful regarding biofilm virulence.

Considering oral microbiota modulation and the consequent biofilm accretion in the presence of sucrose for microcosm biofilm, the data showed that, depending on whether biofilms were exposed during initial formation (from 0 h; adhesion-phase treatment) or after establishment (exposure after 24 h of biofilm formation; 24 h-old biofilm treatment), specific substances interfered with the population of specific microbial groups. Specifically, urea exhibited a similar effect to arginine in hindering the build-up of the initial biofilm, while proline hindered the accretion of preformed biofilms; this effect was not observed for arginine or urea. This effect on initial biofilms could be attributed to the inhibitory effect of arginine on water-insoluble glucan production, which may also interfere with glucan binding, the primary mechanism used by mutans streptococci to attach to their self-produced glucans.^41^ A previous study demonstrated that proline could benefit the growth of saliva-derived biofilms in the absence of sugars, as these biofilms metabolize proline to 5-aminopentanoate, butyrate, and propionate.^24^ Thus, here, in the presence of sucrose, the proline effect may be that it alters bacterial metabolism^27,42^ and interferes with microbial surface characteristics (e.g., cell wall), which, in turn, would affect how microbial cells interact with each other and with extracellular matrix components.

Proline, like arginine, is an amino acid present in saliva and the diet that is not merely a nutrient for oral bacteria; thus, it can serve as a key metabolic substrate (https://www.kegg.jp/pathway/map00330) that shapes the ecological dynamics of dental biofilm communities.^7,46^ Most data on microbial metabolic pathways have been obtained in planktonic culture models; nevertheless, the same pathways are also used by microbial cells in biofilms,^27^ and cells may have switched on genes that were off in the planktonic growth phase, and vice versa. Thus, it is important to consider the global metabolic traits of cells in microcolonies within biofilms. Hence, in this context, the effects of proline could be related to Stickland reactions,^42^ as demonstrated by proline reduction to δ-amino-*n*-valerate in dental plaque samples from monkeys, and this activity was coupled with certain end products of bacterial glucose metabolism.^42^ Thus, at the time, it was postulated that the bound and free proline in the oral environment could be involved in base production to counteract the acid production after host sugar ingestion.^41^ However, besides this important role, and since there was less biomass for mixed-species and the 24 h-old microcosm biofilms, coupled with fewer mutans streptococci in the two models of microcosm biofilms, proline may hinder microcolony development in those biofilms, since there was less area occupied by the smaller microcolonies in the mixed-species biofilm (Figures 6 and 10B).

The urea effect on initial biofilms could be because of interference with glucan binding, coupled with lower counts of aciduric microbiota and fungi in the microcosm biofilm, suggesting that urea may aid in controlling both aciduric organisms and fungi (many of which are aciduric) in oral biofilms.^43^ Thus, these data show the potential of urea as a prebiotic. One may wonder why urea has not been used in oral healthcare products, given its relatively low cost. One drawback may be that it promotes the growth of microbial species associated with an alkalinized milieu, which may be problematic for individuals prone to developing gingivitis, periodontal diseases, and possibly peri-implant diseases. However, there is little evidence to support this hypothesis.^27,44^ Another factor may be that higher concentrations of urea and arginine (20 mM or 0.12% and 0.35%, respectively) induce germ tube formation in ≥80% of the *Candida albicans* strain A72 (ATCC MYA-2430) cells but lower concentrations (5 mM or 0.024% and 0.07%, respectively) induce germ tubes in about 30% of the cells exposed to these substances, and this effect is mediated by a cell-density-dependent pathway.^45^ Also, the combination of urea and miconazole has a synergic effect on *C. albicans*.^46^ Thus, additional research is needed to pinpoint the effect of urea and arginine in controlling fungal population in the polymicrobial oral microbiota, as here, the concentration used was higher than in previous studies (1.5%).

Here, the concentration of arginine was based on the oral care products available and tested in clinical trials^16–18^ and also tested experimentally. The concentrations of urea, proline, sodium nitrate, and GlcNAc were determined experimentally, taking into account concentrations used in previous studies.^21,22,24–26^ Of note, urea is present in saliva and in gingival crevicular fluid at concentrations similar to those in the serum (0.06 to 0.6%).^34^ In contrast, arginine is present in low quantities in saliva but is abundant in the peptide form;^34^ as is proline, as it is derived from the breakdown of proline-rich proteins (PRPs),^47^ the most abundant proteins in saliva. GlcNAc and sodium nitrate levels may vary, as they result from broken down bacterial cells and dietary sources, respectively. Therefore, oral care adjuvant formulations can be created with specific concentrations designed to modulate the microbiota within the biofilms.

Among the tested substances, nitrate affected the size and shape of microcolonies of *S. mutans* and the mixed-species biofilm. A previous work has shown that nitrate/nitrite substrates with glucose reduced acidification,^48^ which could interfere with the population of acidogenic/aciduric microorganisms in biofilms; however, this effect was not observed in the present study. Nevertheless, altering the 3D structure of microcolonies and, consequently, biofilms can impact the diffusion of substances in and out of these structures, even if the ratio of bacteria biovolume to exopolysaccharides is close to one because the 3D structure interferes with the distance and net charge for diffusion. It is well-established that extracellular insoluble glucans present in cariogenic biofilms, along with their 3D configuration, can hinder the diffusion of substances, thereby creating acidic niches within the tridimensional structure of microcolonies.^14^

Regarding *N*-acetylglucosamine (GlcNAc), the current data differ from the previously reported data because we used 0.2212% (10 mM) GlcNAc in the presence of 1% sucrose (29.21 mM), which was used at a much lower concentration in the assays performed in the previous study (2 mM sucrose with 20 mM GlcNAc^21^). Also, another study used 0.9954% (45 mM) GlcNAc and did not use sucrose in the biofilm model.^23^ One hypothesis is that GlcNAc could be easily metabolized (catabolized) to promote *S. mutans* growth. At the same time, the concomitant sucrose could serve primarily for exopolysaccharide production, as GlcNAc could be taken up by PTS (PTS-dependent transport of GlcNAc^22^) and perhaps other sugar transport systems, while sucrose may affect the extracellular carbohydrate metabolism. In addition to these processes, the bacterial cell surface may be affected by cell–wall turnover, which, in turn, could alter how extracellular matrix components connect to bacterial cells in biofilms, thereby altering biofilm architecture. So, GlcNAc may be promoting the fitness of *S. mutans* in the mixed-species model containing *S. gordonii*, as *S. mutans* counts are higher than *S. gordonii*.

A limitation of this research was the use of in vitro biofilm models that do not permit the assessment of demineralization and lack sequencing-based methods to evaluate the microbiota/microbiome. However, current outcomes can help select substances for further investigation in more complex and clinically relevant models to examine how microbial responses to putative prebiotic substances influence demineralization in in situ and/or clinical studies, in tandem with the characterization of the microbiome using sequencing-based methodology. Nevertheless, a challenge in controlling biofilms is to implement strategies that do not trigger antimicrobial resistance or tolerance or select microbial species that are more prone to eliciting this issue. Thus, it may be necessary to tailor and combine strategies to promote health-related biofilms. For example, the mechanism by which these putative prebiotics may interact with fluoride to modulate biofilms and prevent demineralization remains unknown, and this topic warrants further investigation. Also, whether there are potential side effects or considerations for using putative prebiotics in oral healthcare products still warrants further investigation.

Another point is that at 0 h, the three species together were exposed to a lower amount of the tested substances in the mixed-species biofilms than *S. mutans* cells in single-species biofilms. In more detail, for GlcNAc, we used 0.2212% (0.002212 g/mL or 2.2121 mg/mL). So, for *S. mutans* in the single-species biofilm, 2 × 10^6^ CFU/mL were exposed to 2.212 mg/mL GlcNAc. In contrast, for mixed-species (*S. mutans*, *S. gordonii*, and *A. naeslundii*) biofilms, there were 6 × 10^6^CFU/mL (2 × 10^6^CFU/mL per species) that were exposed to 2.212 mg/mL GlcNAc. For arginine, proline, and urea, we used 1.5% (0.015 or 15 mg/mL). So, for *S. mutans* in the single-species biofilm, 2 × 10^6^CFU/mL were exposed to 15 mg/mL arginine, proline, or urea. In contrast, for mixed-species (*S. mutans*, *S. gordonii*, and *A. naeslundii*) biofilms, there were 6 × 10^6^ CFU/mL (2 × 10^6^ CFU/mL per species) exposed to 15 mg/mL arginine, proline, or urea. For NaNO_3_, we used 0.0553% (0.00553 g/mL or 5.53 mg/mL). So, for *S. mutans* in a single-species biofilm, the 2 × 10^6^ CFU/mL were exposed to 5.53 mg/mL NaNO_3_. In contrast, for mixed-species (*S. mutans*, *S. gordonii*, and *A. naeslundii*) biofilms, there were 6 × 10^6^ CFU/mL (2 × 10^6^ CFU/mL per species) exposed to 5.53 mg/mL NaNO_3_. Nevertheless, a single-species *S. mutans* biofilm was used because it produces copious amounts of insoluble glucans, a virulence determinant in cariogenic biofilms, and a model for proof-of-concept assays.

Traditionally, prebiotics have been defined as substances—primarily dietary fibers—that modulate the composition and activity of the gut microbiota. However, in the oral cavity, microorganisms must adhere to surfaces and grow as structured biofilms to persist, as they are continuously exposed to mechanical stresses such as speaking and mastication.^49^ Consequently, a putative oral prebiotic must interfere with the biofilm lifestyle of oral microorganisms rather than merely impeding microbial growth. In this context, the 2017 international consensus defined a prebiotic as “a substrate that is selectively utilized by host microorganisms, conferring a health benefit”.^50^ This updated definition broadened the concept of prebiotics to include noncarbohydrate substances, applications beyond the gastrointestinal tract, and categories other than food.^50,51^

In the context of dental caries, substances that generate alkali—such as arginine and urea— are degraded by certain oral bacteria, leading to the formation of ammonia and an increase in oral pH. Although research has not demonstrated that adding urea to chewing gum or mouthwash significantly reduces caries risk,^19^ numerous studies indicate that arginine-containing consumer products can inhibit caries development.^16–19^ How the microbial metabolism of proline or sodium nitrate can modulate pathways linked to microbial growth per se or extracellular factors linked to biofilm build-up does need further investigation. In this study, the tested substances may not have affected microbial growth per se but modulated biofilm development, including biofilm biomass and microbial population structure, contributing to the modulation of the species present, as indicated by the culture-based approaches using selective media. However, these methods do not fully capture the complexity of microbial community dynamics or directly demonstrate selective substrate utilization. Therefore, while the findings suggest a shift in functionally relevant microbial populations, they should be interpreted as an indicative of ecological modulation rather than definitive evidence of selective prebiotic activity. Nevertheless, the microbial community in microcosm biofilms was investigated using a culturing method that limited the groups of microorganisms evaluated; hence, further investigations should employ sequencing-based methods^25^ to fully assess the shift in microorganisms present in biofilms subjected to putative prebiotic substances or formulations with the combination of distinct substances that could target distinct microbial pathways.

## Conclusions

The use of prebiotic substances could be a strategy to help control cariogenic biofilms.^52^ However, the choice of substances needs to be tailored to account for a multitarget approach to avoid dysbiosis, as some of the findings presented here differ from those of previous studies.^21–26^ In the current study, besides arginine, proline, and urea may also function as prebiotics to modulate the microbial population and its products linked to cariogenicity (e.g., glucan binding and consequently the 3D architecture linked to diffusion^41^), potentially serving as an adjuvant measure to control cariogenic biofilms and promote homeostasis to prevent microbial ecological imbalances. In contrast, sodium nitrate may not be suitable as a prebiotic for controlling cariogenic biofilms in vitro, but it could still be further tested in more clinically relevant models. Thus, these findings underscore the potential of prebiotics in oral health strategies to manage oral biofilm-related diseases.

## Data Availability

The data supporting this study are available within the manuscript.

## §

Anhanguera College of Piracicaba, Piracicaba, São Paulo 13425-075, Brazil
